# Predictors of modern contraceptive use among adolescent girls and young women in sub-Saharan Africa: a mixed effects multilevel analysis of data from 29 demographic and health surveys

**DOI:** 10.1186/s40834-020-00138-1

**Published:** 2020-11-19

**Authors:** Bright Opoku Ahinkorah

**Affiliations:** School of Public Health, Faculty of Health, University of Technology Sydney, Sydney, Australia

**Keywords:** Utilization of modern contraceptives, Adolescent girls, Young women, Sub-Saharan Africa, Reproductive health

## Abstract

**Background:**

The use of modern contraceptives among adolescent girls and young women (AGYW) in sub-Saharan Africa (SSA) remains an issue that needs urgent attention. This present study assesses the individual and contextual factors associated with modern contraceptive use among AGYW in SSA.

**Methods:**

Data for this study was obtained from the latest Demographic and Health Surveys (DHS) conducted between January 2010 and December 2018 across 29 countries in SSA. Data were analysed with Stata version 14.2 by employing both Pearson’s chi-square test of independence and a multilevel binary logistic regression. The selection of variables for the multilevel models was based on their statistical significance at the chi-square test at a *p* < 0.05. Model fitness for the multilevel models was checked using the log likelihood ratios and Akaike’s Information Criterion (AIC) and the results were presented using adjusted odds ratios (aOR) at 95% confidence interval (CI).

**Results:**

It was found that 24.7% of AGYW in SSA use modern contraceptives. In terms of the individual level factors, the study showed that AGYW aged 15–19 [aOR = 0.86, CI = 0.83–0.90], those who were married [aOR = 0.83, CI = 0.79–0.87], Muslims [aOR = 0.59, CI = 0.57–0.62], working [aOR = 0.92, CI = 0.89–0.95], those who had no child [aOR = 0.44, CI = 0.42–0.47], those who had no exposure to newspaper/magazine [aOR = 0.44, CI = 0.63–0.71] and radio [aOR = 0.82, CI = 0.78–0.86] had lower odds of using modern contraceptives. Conversely, the use of modern contraceptives was high among AGYW whose age at first sex was 15–19 years [aOR = 1.20, CI = 1.12–1.28]. With the contextual factors, the odds of using modern contraceptives was low among AGYW who lived in rural areas [aOR = 0.89, CI = 0.85–0.93] and in communities with low literacy level [aOR = 0.73, CI = 0.70–0.77] and low socio-economic status [aOR = 0.69, CI = 0.65–0.73].

**Conclusion:**

Several individual and contextual factors are associated with modern contraceptive use among AGYW in SSA. Therefore, Governments in the various countries considered in this study should intensify mass education on the use of modern contraceptives. This education should be more centered on AGYW who are in socio-economically disadvantaged communities, those who are not married, Muslims, those with high parity and high fertility preferences and those who are working.

## Background

The sexual and reproductive health (SRH) of adolescent girls and young women (AGYW) aged 15–24 forms a key component of the global burden of sexual ill health [1]. This is because neglecting SRH of AGYW has detrimental effect on their transition to adulthood [2]. The SRH of AGYW has become critical because it has been estimated that 16 million girls aged 15–19 give birth each year, and this constitutes about 11% of all births worldwide and 95% of these births occur in low-and middle-income countries (LMICs), including sub-Saharan Africa (SSA) [3]. Other studies have indicated that most of these births result from unintended pregnancies and in SSA, 44% of these unintended pregnancies occur among AGYW [4, 5]. This unquestionably accounts for the high unsafe abortion rates among AGYW in SSA [6]. Apart from these SRH challenges, AGYW in SSA also face risk of exposure to HIV and other sexually transmitted infections (STIs) [1].

As part of efforts to deal with the SRH challenges of AGYW, the United Nations in 2015 developed the Sustainable Development Goals and Goal 3.7 focuses on ensuring universal access to sexual and reproductive health-care services, including family planning, information and education, and the integration of reproductive health into national strategies and programmes by 2030 [7]. The achievement of this Goal, especially for AGYW who often go through a lot of SRH challenges depends on the access, consistent and effective use of modern contraceptives [8, 9].

However, studies have indicated that the use of modern contraceptives among AGYW in SSA remains an issue that needs urgent attention [10–12]. Globally, it is estimated that over 220 million women in LMICs have an unmet need for contraception [13]. Although the prevalence of unmet need for contraception among AGYW in SSA is unknown, approximately 25% of women in the subregion (i.e. about 47 million) have unmet need for contraception [14], with a majority of them being AGYW [15–17].

Theoretically, the use of modern contraceptives among AGYW can be understood within the theoretical underpinning of the Health Belief Model which provides explanations to the likelihood of using modern contraceptives among AGYW from the point of perceived risks [18]. In this regard, AGYW who consider themselves as being at risk of unintended pregnancies and STIs may be more likely to use modern contraceptives compared to those who consider themselves not to be at risk. In the absence of risk perception, studies have shown that despite the desire of AGYW to use contraceptives, a majority of them have challenges with access to contraceptive services [15–17]. These challenges can be understood within the context of Anderson and Newmans’ Health Care Utilisation Model which proposes that the use of a service, which includes modern contraceptives is influenced by predisposing factors such as demographics, health beliefs and social structures; enabling factors which include the availability of health personnel and facilities, waiting time and health insurance subscription and need for care factors which focuses on factors which are people’s perception and evaluation of their health that serve as motivation to use a service [19].

Based on these theoretical underpinnings, it can be affirmedthat individual and contextual factors play a key role in the use of modern contraceptives among AGYW. However, studies on modern contraceptives use among AGYW in SSA have often focused on individual level factors such as age, marital status, religion, ethnicity, level of education, wealth status and occupation [17, 20–23]. Apart from their focus on individual level factors, most of these studies examined the predictors of contraceptive use (which includes traditional methods) instead of looking at modern contraceptives which have been considered the most effective of all contraceptive methods [24–26]. Again, studies on predictors of contraceptives among AGYW in SSA have been done in specific countries like Congo [20], Ghana [23] Malawi [21], South Africa [17] and Tanzania [22]. Although a recent study explored the predictors of modern contraceptive use among young women in LMICs, which included countries in SSA, the major focus of the authors was on community level factors that influence modern contraceptive use [27]. One of the gaps in this study which the current study seeks to fill is the lack of understanding of the interaction between individual and contextual-level factors in influencing the use of modern contraceptives of among AGYW. Again, Mutumba, Wekesa [27] used data from the 2008 to 2016 Demographic and Health Surveys (DHS). However, the current study considers current DHS data from 2010 and 2018 since variations in contraceptive accessibility and usage may have happened in the last few years. Using a multilevel modelling approach, this present study assesses the individual and contextual factors associated with modern contraceptive use among AGYW in SSA. Findings from this study will help to formulate useful interventions and strategies in addressing the use of modern contraceptive among AGYW in the sub-region.

## Methods

### Data source

Data for this study was obtained from the latest DHS conducted between January 2010 and December 2018 across 29 countries in SSA. The survey was designed to collect and provide data on various demographic indicators such as contraceptive use [28]. The details of the methodology employed in the DHS are documented by Corsi, Neuman [29]. In this study, only AGYW (15–24 years) who had ever had sex and had information on the use of contraceptives were considered (*n* = 91,083). Table 1 gives detailed description of the countries, survey years and the sample used in this study. The datasets for the DHS are available at http://dhsprogram.com/data/available-datasets.cfm.

**Table 1:** Detailed description of the countries, survey years and the sample used in this study

Country	Survey year	Adolescent girls and young women	Sample (N)	Sample (%)
1. Angola	2015–16	6423	4822	5.3
2. Benin	2017–18	6251	4084	4.5
3. Burkina Faso	2010	6592	4319	4.7
4. Burundi	2016–17	7218	2579	2.8
5. Cameroon	2011	6708	4187	4.6
6. Chad	2014–15	6884	3374	3.7
7. Comoros	2012	2282	683	0.8
8. Congo	2011–12	3963	3235	3.6
9. Côte d’Ivoire	2011–12	3984	2814	3.1
10. DR Congo	2013–14	7661	4953	5.4
11. Ethiopia	2016	6401	2625	2.9
12. Gabon	2012	3407	2487	2.7
13. Gambia	2013	4564	1875	2.1
14. Ghana	2014	3327	2046	2.3
15. Guinea	2018	4267	2315	2.5
16. Kenya	2014	11,483	3215	3.5
17. Lesotho	2014	2842	589	0.7
18. Liberia	2013	3499	2921	3.2
19. Malawi	2015–16	10,369	7382	8.1
20. Mali	2018	4116	2649	2.9
21. Namibia	2013	3577	2151	2.4
22. Nigeria	2018	15,267	4740	5.2
23. Rwanda	2014–15	5252	2084	2.3
24. Senegal	2010–11	6773	2433	2.7
25. Sierra Leone	2013	6739	3059	3.4
26. Togo	2013–14	3337	2188	2.4
27. Uganda	2016	8058	5303	5.8
28. Zambia	2018	5799	3832	4.2
29. Zimbabwe	2015	3938	2138	2.4

### Definition of variables

#### Outcome variable

The outcome variable for the study was ‘current modern contraceptive usage’. It was derived from the question, “Are you currently using any type of contraceptive”? Responses to this question were coded as “no method”, “folkloric method”, “traditional method” and “modern method”. Details of the specific contraceptives under each of the methods have been described elsewhere [30]. The existing DHS variable excluded women who were pregnant and those who had never had sex. For the purpose of this study, AGYWwho were using modern methods were coded as ‘1’ whiles those who were not using any methods, those using traditional methods and folkloric methods were recoded as ‘0’ [31, 32].

#### Independent variables

Fifteen independent variables, grouped into individual and contextual level factors were considered in this study. These variables were not determined a priori; but were selected based on their theoretical relevance and practical significance with usage of modern contraceptives [26, 33–35].

#### Individual level factors

The individual level factors were age, marital status, religion, employment status, age at first sex, parity, frequency of reading newspaper/magazine, listening to radio and watching television, desire for more children and ideal number of children. Age was coded as ‘15–19’ and ‘20–24’. Marrital status was recoded into ‘never married’, ‘married’, ‘cohabiting’ and ‘widowed/divorced/separated’. Religion was recoded as ‘Christianity’, ‘Islam’ and ‘other’. ‘Working’ and ‘not working’ were the categories for employment status. Age at first sex was recoded as ‘less than 15 years’ ‘15–19 years’ and ‘20–24 years’. Parity was recoded as ‘zero birth’, ‘one birth’, ‘two births’, ‘three births’, and four or more births’. Frequency of reading newspaper/magazine, listening to radio and watching television were each coded as ‘not at all’ ‘less than once a week’ and ‘at least once a week’. ‘Desire for more children and idela number of children were derived from the questions, “do you desire to have a/another child?” and “what is the ideal number of children you want to have?”. For this study, desire for more children was coded as “have another”, “undecided” and “no more”. Ideal number of children was also coded as “0–3″, “4–5″ and 6+”.

#### Community level factors

Residence, community literacy level (proportion of women who can read and write), community socio-economic status (proportion of women in the richest household quintile) and community knowledge level of modern contraceptive (proportion of women with knowledge on modern contraceptives) were considered as contextual level factors. Residence was coded as ‘urban’ and ‘rural’. Community literacy level, community socio-economic status and community knowledge level of modern contraceptives were each coded as ‘low’, ‘middle’ and ‘high’.

### Statistical analyses

The data were analysed with Stata version 14.2 for windows following a three-step analytical approach. The first approach involved the use of percentages to describe the prevalence of modern contraceptive use among AGYW in SSA. This was followed by the distribution of modern contraceptive use across the individual and contextual level factors. Statistical significance of the association between each of the factors and modern contraceptive use was measured using Pearson’s chi-square test of independence [χ^2^] at a *p*-value of less than 0.05 (see Table 1). Finally, a two-level multilevel binary logistic regression analysis was carried out to examine the association between modern contraceptive use and the individual and contextual factors. The use of a multilevel binary logistic regression in this study was to cater for the different levels at which sampling was done for the DHS (individual, household and cluster levels). The two-level modelling in this study implies that women were nested within households and households were nested within clusters. Clusters were considered as random effects to cater for the unexplained variability at the contextual level [36]. Only variables that showed statistically significant associations with modern contraceptive utilization at the chi-square test were included in the multilevel binary logistic regression models.

In terms of the modelling, four models, comprising the empty model (model 0), model 1 model 2 and model 3 were fitted. Model 0 showed the variance in modern contraceptive use attributed to the clustering of the primary sampling units (PSUs) without the explanatory variables. Model 1 and model 2 contained the individual and contextual level factors respectively while model 3 contained all the individual and contextual level factors. The Stata command “melogit” was used in fitting these models. Model comparison was done using the log likelihood and Akaike’s Information Criterion (AIC) tests. The lowest AIC (90,917.38) and highest log likelihood (−45,426.69) were used to determine the best fit model (see Table 2). Odds ratio and associated 95% confidence intervals (CIs) were presented for all the models apart from model 0 (see Table 2). To check for high correlation among the explanatory variables, a test for multicollinearity was carried out using the variance inflation factor (VIF) and the results showed no evidence of high collinearity (Mean VIF = 1.35, Maximum VIF = 1.86, and Minimum VIF = 1.06). Sample weight (v005/1,000,000) and SVY command were used to correct for over and under-sampling and the complex survey design and generalizability of the findings respectively. The findings were presented using the Strengthening Reporting of Observational studies in Epidemiology (STROBE) reporting guidelines [37].

**Table 2:** Distribution of modern contraceptive use across individual and contextual factors of adolescent girls and young women in sub-Saharan Africa

Variables	Weighted N	Weighted %	Modern contraceptive use	χ2 (***p***-value)
**Age**				365.7 (< 0.001)
15–19	33,197	36.4	21.1	
20–24	57,886	63.6	26.8	
Marital status				476.9 (< 0.001)
Never married	33,359	36.6	28.5	
Married	38,564	42.3	21.3	
Cohabiting	14,012	15.4	24.6	
Widowed/divorced/separated	5148	5.7	25.7	
**Religion**				8.2 (< 0.001)
Christianity	60,928	66.9	29.1	
Islam	25,992	28.5	15.0	
Other	4162	4.6	20.5	
**Employment status**				45.3 (< 0.001)
Not working	37,956	41.7	26.2	
Working	53,127	58.3	23.6	
**Age at first sex**				281.8 (< 0.001)
Less than 15 years	19,252	21.1	20.1	
15–19 years	65,236	71.6	25.9	
20–24 years	6595	7.2	26.2	
**Parity**				371.0 (< 0.001)
No birth	33,799	37.1	22.3	
One birth	31,748	34.9	27.0	
Two births	16,884	18.5	27.6	
Three births	6577	7.2	21.7	
Four or more births	2074	2.3	14.7	
**Frequency of reading newspaper/magazine**				7.9 (< 0.001)
Not at all	70,291	77.2	21.4	
Less than once a week	10,880	12.0	35.4	
At least once a week	9912	10.9	36.5	
**Frequency of listening to radio**				698.1 (< 0.001)
Not at all	36,24	40.1	20.5	
Less than once a week	17,828	19.6	26.0	
At least once a week	36,731	40.3	28.3	
**Frequency of watching television**				640.2 (< 0.001)
Not at all	48,953	53.8	21.6	
Less than once a week	11,640	12.8	25.9	
At least once a week	30,490	33.5	29.3	
**Desire for more children**				263.2 (< 0.001)
Have another	81,348	89.3	24.4	
Undecided	3668	4.0	20.1	
No more	6067	6.7	31.7	
**Ideal number of children**				12.5 (< 0.001)
0–3	28,448	31.2	33.7	
4–5	41,582	45.7	25.6	
6+	21,052	23.1	10.8	
**Place of residence**				969.0 (< 0.001)
Urban	37,290	40.9	30.0	
Rural	53,793	59.1	21.0	
**Community literacy level**				3.3 (< 0.001)
Low	29,156	32.0	18.1	
Moderate	33,323	36.6	27.0	
High	28,603	31.4	28.8	
**Community socio-economic status**				7.3 (< 0.001)
Low	50,072	55.0	19.9	
Moderate	7563	8.3	30.7	
High	33,448	36.7	30.5	
**Community knowledge of modern method**				6.0 (< 0.001)
Low	72,827	78.0	27.1	
Moderate	16,786	18.4	15.6	
High	1470	1.6	9.5	

Source: Demographic and Health Surveys

### Ethical approval

Ethical clearance were obtained from the Ethics Committee of ORC Macro Inc. as well as Ethics Boards of partner organisations of the various countries such as the Ministries of Health. The DHS follows the standards for ensuring the protection of respondents’ privacy. Inner City Fund (ICF) International ensured that the survey complies with the U.S. Department of Health and Human Services regulations for the respect of human subjects. This was a secondary analyses of data and therefore no further approval was required since the data is available in the public domain. Further information about the DHS data usage and ethical standards are available at http://goo.gl/ny8T6X.

## Results

**Fig. 1:**
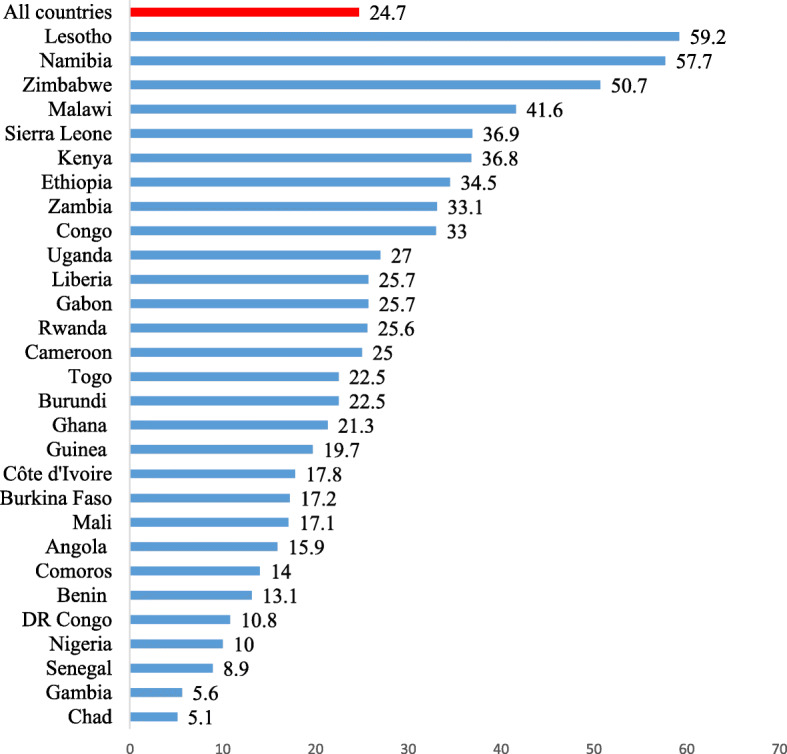
Prevalence of modern contraceptive usage among adolescent girls and young women in SSA. Source: Demographic and Health Surveys

Figure 1 displays the results on the prevalence of modern contraceptive use among AGYW in SSA. It was found that 24.7% of AGYW in the 29 countries in SSA considered in this study use modern contraception. Lesotho recorded the highest prevalence of modern contraceptive use among AGYW (59.2%) while Chad had the lowest prevalence of 5.1%.

### Distribution of modern contraceptive use across the individual and contextual level factors

Table 2 shows results on the distribution of modern contraceptive use across the individual and contextual level factors of AGYW in SSA. The results indicate that the use of modern contraceptives was high among AGYW aged 20–24 (26.8%), those who had never married (28.5%), Christians (29.1%), those who were not working (26.2%), those who had their first sex between 20 and 24 years (26.2%), those with two births (27.6%) and those who read newspaper/magazine, listened to radio or watched television at least once a week. A greater proportion of AGYW also used modern contraceptives if they desired to have no more children (31.7%), had 0–3 as their ideal number of children (33.7%), lived in urban areas (30%), lived in communities with high literacy (28.8%), moderate socio-economic status (30.7%) and low knowledge of modern methods (27.1%). The chi-square test results also revealed all the individual and contextual factors had statistically significant associations with modern contraceptive usage.

### Association between individual and contextual level factors and modern contraceptive use among AGYW in SSA

In terms of the individual level factors, the study showed that AGYW aged 15–19 were less likely to use modern contraceptives [aOR = 0.86, CI = 0.83–0.90] compared with those aged 20–24. AGYW who were either married [aOR = 0.83, CI = 0.79–0.87], cohabiting [aOR = 0.69, CI = 0.66–0.73], or widowed/divorced/separated [aOR = 0.77, CI = 0.72–0.83] had lower odds of using modern contraceptives, compared with those who had never married. The likelihood of modern contraceptive use was also low among Muslims [aOR = 0.59, CI = 0.57–0.62] and AGYW who belonged to ‘other’ religions [aOR = 0.68, CI = 0.62–0.74] compared to Christians. AGYW who were working [aOR = 0.92, CI = 0.89–0.95], those who had no child [aOR = 0.44, CI = 0.42–0.47], those who had no exposure to newspaper/magazine [aOR = 0.44, CI = 0.63–0.71] and radio [aOR = 0.82, CI = 0.78–0.86] had lower odds of using modern contraceptives. The likelihood of modern contraceptive use among AGYW reduced among those who were not decided on their desire for more children [aOR = 0.72, CI = 0.65–0.80] and those whose ideal number of children was 6+ [aOR = 0.38, CI = 0.35–0.40]. Conversely, the use of modern contraceptives was high among AGYW whose age at first sex was 15–19 years [aOR = 1.20, CI = 1.12–1.28] compared to those whose first sex happened between the ages of 20–24. With the contextual factors, the odds of using modern contraceptives was low among AGYW who lived in rural areas [aOR = 0.89, CI = 0.85–0.93], communities with low literacy level [aOR = 0.73, CI = 0.70–0.77], low socio-economic status [aOR = 0.69, CI = 0.65–0.73] and high knowledge of modern contraceptives [aOR = 0.33, CI = 0.27–0.41] compared to those who lived in urban areas, communities with high literacy level, high socio-economic status and low knowledge on modern contraceptives (see Table 3).

**Table 3:** Mixed effects results on individual and contextual factors associated with modern contraceptive use among adolescent girls and young women in sub-Saharan Africa

Variables	Model 0 aOR[95%CI]	Model 1 aOR[95%CI]	Model 2 aOR[95%CI]	Model 3 aOR[95%CI]
**Age**				
15–19		0.82^***^ (0.79–0.86)		0.86^***^ (0.83–0.90)
20–24		1		1
**Marital status**				
Not married		1		1
Married		0.80^***^ (0.76–0.83)		0.83^***^ (0.79–0.87)
Cohabiting		0.68^***^ (0.65–0.72)		0.69^***^ (0.66–0.73)
Widowed/separated/divorced		0.78^***^ (0.72–0.84)		0.77^***^ (0.72–0.83)
**Religion**				
Christianity		1		1
Islam		0.58^***^ (0.55–0.60)		0.59^***^ (0.57–0.62)
Other		0.69^***^ (0.63–0.75)		0.68^***^ (0.62–0.74)
**Employment status**				
Not working		1		1
Working		0.89^***^ (0.86–0.92)		0.92^***^ (0.89–0.95)
**Age at first sex**				
Less than 15 years		0.99 (0.91–1.06)		1.03 (0.95–1.11)
15–19 years		1.18^***^ (1.11–1.26)		1.20^***^ (1.12–1.28)
20–24 years		1		1
**Parity**				
Zero birth		0.48^***^ (0.45–0.50)		0.44^***^ (0.42–0.47)
One birth		0.78^***^ (0.75–0.82)		0.76^***^ (0.72–0.80)
Two births		1		1
Three births		0.92^*^ (0.85–0.98)		0.94 (0.87–1.01)
Four or more births		0.85^**^ (0.75–0.96)		0.88 (0.77–1.00)
**Frequency of reading newspaper/magazine**				
Not at all		0.65^***^ (0.61–1.82)		0.67^***^ (0.63–0.71)
Less than once a week		1.02 (0.96–1.08)		0.98 (0.91–1.04)
At least once a week		1		1
**Frequency of listening to radio**				
Not at all		0.80^***^ (0.77–0.83)		0.82^***^ (0.78–0.86)
Less than once a week		1.00 (0.95–1.04)		0.99 (0.95–1.04)
At least once a week		1		1
**Frequency of watching television**				
Not at all		0.85^***^ (0.81–0.88)		0.97 (0.93–1.01)
Less than once a week		0.92^**^ (0.88.0.97)		1.00 (0.95–1.06)
At least once a week		1		1
**Desire for more children**				
Have another		1.10^**^ (1.03–1.17)		1.08^*^ (1.01–1.15)
Undecided		0.70^***^ (0.63–0.77)		0.72^***^ (0.65–0.80)
No more		1		1
**Ideal number of children**				
0–3		1		1
4–5		0.73^***^ (0.70–0.75)		0.78^***^ (0.75–0.81)
6+		0.31^***^ (0.29–0.32)		0.38^***^ (0.35–0.40)
**Place of Residence**				
Urban			1	1
Rural			0.83^***^ (0.79–0.86)	0.89^***^ (0.85–0.93)
**Community literacy level**				
Low			0.63^***^ (0.60–0.66)	0.73^***^ (0.70–0.77)
Moderate			0.98 (0.94–1.02)	0.95^*^ (0.92–0.99)
High			1	1
**Community socio-economic status**				
Low			0.66^***^ (0.63–0.70)	0.69^***^ (0.65–0.73)
Moderate			1	1
High			0.96 (0.91–1.02)	0.90^***^ (0.84–0.95)
**Community knowledge of modern method**				
Low			1	1
Moderate			0.43^***^ (0.40–0.44)	0.53^***^ (0.51–0.56)
High			0.24^***^ (0.19–0.29)	0.33^***^ (0.27–0.41)
**Random effect result**				
PSU variance (95% CI)	0.03 (0.02–0.04)	0.02 (0.01–0.03)	0.03 (0.01–0.04)	0.02 (0.01–0.03)
ICC	0.008	0.006	0.007	0.006
LR Test	χ2 = 91.46, *p* < 0.001	χ2 = 47.41, p < 0.001	χ2 = 68.69, p < 0.001	χ2 = 45.92, p < 0.001
Wald chi-square	Reference	6019.62^***^	3323.50^***^	7015.95^***^
**Model fitness**				
Log-likelihood	−49,667.17	−46,122.51	−47,848.36	−45,426.69
AIC	99,338.16	92,295.02	95,714.72	90,917.38
N	91,083	91,083	91,083	91,083

Source: Demographic and Health Surveys

1 Reference category, *PSU* Primary Sampling Unit, *ICC* Intra-Class Correlation, *LR Test* Likelihood ratio Test, *AIC* Akaike’s Information Criterion

^*^p < 0.05, ^**^p < 0.01, ^***^p < 0.001

## Discussion

This study assessed the individual and contextual level correlates of modern contraceptive use among AGYW in SSA. It was found that 24.7% of AGYW in SSA use modern contraceptives. Age, marital status, religion, employment status, parity, exposure to mass media, desire for more children, ideal number of children and age at first sex were identified as individual level predictors of modern contraceptive use among AGYW in SSA. In terms of the contextual level factors, place of residence, community literacy level, community socio-economic status and community knowledge of modern contraceptives had associations with the utilization of modern contraceptives among AGYW in SSA.

In this study, the use of modern contraceptives was low among AGYW in SSA. In line with this finding, Mutumba, Wekesa [27] also found a low prevalence of 17.8% of modern contraceptive use among young women in LMICs. AGYW in chad had the lowest prevalence of modern contraceptive use. In a recent study on contraceptive use among adolescents and adult women in LMICs, Li, Patton [38] identified Chad as one of the countries with a low contraceptive prevalence. AGYW in Chad are less likely to use modern contraceptives because in Chad, national guidelines authorized only doctors based in hospitals to provide modern contraceptives such as IUDs and implants. Again, few providers in the country have been given the training to provide contraceptive services. This often results in the lack of skills and confidence to deliver these services [39]. The cost of modern contraceptive services has also been considered as a barrier to the use of modern contraceptives, especially among AGYW in Chad [39].

This study showed that AGYW aged 15–19 were less likely to use modern contraceptives compared with those aged 20–24. This finding corroborates the findings obtained in previous studies [20, 21]. The possible reason for this finding is that AGYW aged 20–24 are assumed to have a better understanding of the consequences of engaging in sexual acts without contraception compared to those aged 15–19 [21]. AGYW aged 15–19 may also have challenges accessing family planning services either because they have no knowledge on where to obtain contraception or cannot afford the services [40, 41]. Relatedly, this study identified a high usage of modern contraceptives among AGYW who had their first sex between the ages of 15–19 and those who had never married. The possible reason for these findings is the link between marital status and modern contraceptive use. Studies have explained that AGYW who have never married are more likely to use modern contraceptives to prevent unintended pregnancies [42, 43] while those who are married are confronted with pressure to have a child soon after marriage which expose them to pregnancy even if they had the intention to delay pregnancy [44]. This is true, especially among those whose age at first sex occurs below 20 years as this is more likely to occur outside wedlock [45, 46]. The findings are also in line with the theoretical underpinning of the Health Belief Model which explains that AGYW who consider themselves as being at risk of unintended pregnancies and STIs may be more likely to use modern contraceptives compared to those who consider themselves not to be at risk [18].

Muslim AGYW and those who belonged to ‘other’ religions were less likely to use modern contraceptives compared to Christians. This finding is supported by the findings of other studies [47–49]. This may be attributed to differences in religious teachings regarding the use of contraceptives for fertility control [47]. As found in this study, in religions where high parity is desired and fertility preferences are high, there is low likelihood of the use of modern contraceptives. Specifically for Muslim AGYW, a study by Abdi, Okal [50] in two Muslim communities in Kenya showed that Muslim women tend to have high desire for more children due to the belief that children are a blessing from God. Thus, to receive more of the blessings God has in stock for them in the form of children, they will be less likely to use modern contraceptives. This finding could be true especially when previous studies have found high prevalence of child marriage among Muslims and other religious adherents compared to Christians [51, 52]. Despite this finding, there is the need for future qualitative research to explore further on why Christian AGYW are more likely to use modern contraceptives compared to Muslims and AGYW who belong to ‘other’ religion. In the meantime, there is the need to formulate and disseminate particular culturally appropriate messages targeting sexually active AGYW on the importance of modern contraceptive use in line with their religious beliefs and practices.

In this study, AGYW who lived in socio-economically disadvantaged communities (rural areas, communities with low literacy level and low socio-economic status) were less likely to use modern contraceptives compared to those who lived in socio-economically advantaged communities. This finding is in agreement with the findings of previous studies [53, 54]. This finding could be attributed to economic empowerment that gives AGYW in socio-economically advantaged communities more economic power to access modern contraceptives as the barriers in terms of cost are catered for [55–57]. Theoretically, AGYW who are in socio-economically disadvantaged communities might not have the perceived behavioural control to utilise modern contraceptives when they are financially not viable to purchase these modern contraceptives [58]. Despite the role of advantaged socio-economic status on the use of modern contraceptives, AGYW who were working had lower odds of using modern contraceptives compared to those who were not working. The possible reason for this finding could be that the majority of non-working AGYW may be in school and hence may consider the use of contraceptives as a means of preventing pregnancy and delaying fertility. Similar findings were obtained in Nigeria [36] and Ghana [59].

Non-exposure to media (radio and newspaper/magazine) decreased the odds of modern contraceptive use among AGYW in this study. This finding relates to the findings of studies in other SSA countries like Ghana [23], Mali [60], Nigeria [61, 62] and Senegal [63]. It can be affirmed that access to mass media is likely to lead to exposure to family planning messages which can bring about change in AGYW’s negative attitude with respect to contraception [61]. Relatedly, Sharma, Ghimire [64] also observed that women who are exposed to family planning messages through health facilities, radio, and television have a greater likelihood to use modern contraceptives in Nepal. However, AGYW who lived in communities with high knowledge on modern contraceptives were less likely to use modern contraceptives. Further research is needed to understand this counter-intuitive finding.

### Strengths and limitations

The use of nationally representative and current datasets in this study and the focus on AGYW in SSA is a major strength in this study. Again, the large sample size, the use of rigorous statistical analysis that considers both individual and contextual level factors associated with modern contraceptive use and the use of validated instruments in the DHS strengthen the validity of findings from the dataset. However, the use of cross-sectional design in the surveys makes it impossible to establish causality with respect to the findings. There is also the possibility of response bias since the issue on contraceptive use may lead to provision of social desirable responses and AGYW may also find it challenging to recall previous events on the use of modern contraception. The differences in survey years can also affect the comparability of the findings since modernization may play a role in the prevalence of modern contraceptives use in more current surveys compared to older ones.

## Conclusion

Modern contraceptive use among the AGYW in SSA remains low and this can result in high rates of unwanted or mistimed pregnancies and risk of contracting STIs including HIV/AIDS. Therefore, Governments in the various countries considered in this study through their policy makers should put in measures that will halt barriers to access and use of modern contraceptives whilst intensifying mass education on modern contraceptive methods. This education should be more centered on AGYW who are in socio-economically disadvantaged communities, those who are not married, Muslims, those with high parity and high fertility preferences and those who are working. Further studies is essential to unearth the reasons why AGYW who live in communities with high knowledge on modern contraceptives are less likely to use modern contraceptives.

## Data Availability

Data for this study were sourced from Demographic and Health surveys (DHS) and available here: http://dhsprogram.com/data/available-datasets.cfm.

## Abbreviations

AGYW: Adolescent girls and young women
AIC: Akaike’s Information Criterion
aOR: Adjusted odds ratios
DHS: Demographic and Health Survey
ICC: Intra-Class Correlation
LR Test: Likelihood ratio Test
PSU: Primary Sampling Unit
SRH: Sexual and reproductive health
SSA: Sub-Saharan Africa
